# Adaptations during Maturation in an Identified Honeybee Interneuron Responsive to Waggle Dance Vibration Signals

**DOI:** 10.1523/ENEURO.0454-18.2019

**Published:** 2019-09-05

**Authors:** Ajayrama Kumaraswamy, Hiroyuki Ai, Kazuki Kai, Hidetoshi Ikeno, Thomas Wachtler

**Affiliations:** 1 Department of Biology II, Ludwig-Maximilians-Universität München, Planegg-Martinsried, 82152, Germany; 2 Department of Earth System Science, Fukuoka University, Fukuoka, 814-0180, Japan; 3 School of Human Science and Environment, University of Hyogo, Himeji, 670-0092, Japan

**Keywords:** adaptation, honeybee, maturation, neuron morphology

## Abstract

Honeybees are social insects, and individual bees take on different social roles as they mature, performing a multitude of tasks that involve multi-modal sensory integration. Several activities vital for foraging, like flight and waggle dance communication, involve sensing air vibrations through their antennae. We investigated changes in the identified vibration-sensitive interneuron DL-Int-1 in the honeybee *Apis mellifera* during maturation by comparing properties of neurons from newly emerged adult and forager honeybees. Although comparison of morphological reconstructions of the neurons revealed no significant changes in gross dendritic features, consistent and region-dependent changes were found in dendritic density. Comparison of electrophysiological properties showed an increase in the firing rate differences between stimulus and nonstimulus periods in foragers compared with newly emerged adult bees. The observed differences in neurons of foragers compared with newly emerged adult honeybees suggest refined connectivity, improved signal propagation, and enhancement of response features possibly important for the network processing of air vibration signals relevant for the waggle dance communication of honeybees.

## Significance Statement

In the darkness of the hive, honeybees inform each other about profitable food sources using stereotypic movements accompanied by specific sound patterns produced by wing-beats. Here we present a study of an identified vibration-sensitive neuron, named DL-Int-1, in the honeybee brain focusing on structural and functional adaptations by comparing data from young, newly emerged adult and mature forager honeybees. We found region-dependent changes in the morphological structure of DL-Int-1 as well as specific changes in its response properties, which suggest an adaptation process during maturation leading to a refinement in network connectivity and improved processing of waggle dance signals in the honeybee brain.

## Introduction

Perception of vibrations and sounds is very important for social insects (Hunt and Richard, 2013) and among them, honeybees are unique in that they use air-borne vibrations for communication (Kirchner, 1997). Among several intra-hive communication behaviors linked to air-borne vibration sensing (Barth et al., 2005; Hunt and Richard, 2013; Nieh, 2010), the waggle dance behavior, which is used to communicate the distance, direction and profitability of food sources, has been extensively studied in the honeybee *Apis mellifera* (von Frisch 1965, 1967; Kirchner and Towne 1994; Brockmann and Robinson, 2007; Hrncir et al., 2011; Couvillon, 2012). Waggle dance behavior consists of alternative repetitions of two movements; a straight onward movement called the “waggle phase” during which honeybees produce air vibrations by oscillating their abdomen from side to side and beating their wings; and a curved movement called the “return phase” during which they return to the starting point of the onward phase. The neural mechanisms underlying the processing and decoding of the waggle dance vibration signals have so far not been uncovered.

**Figure 1.**
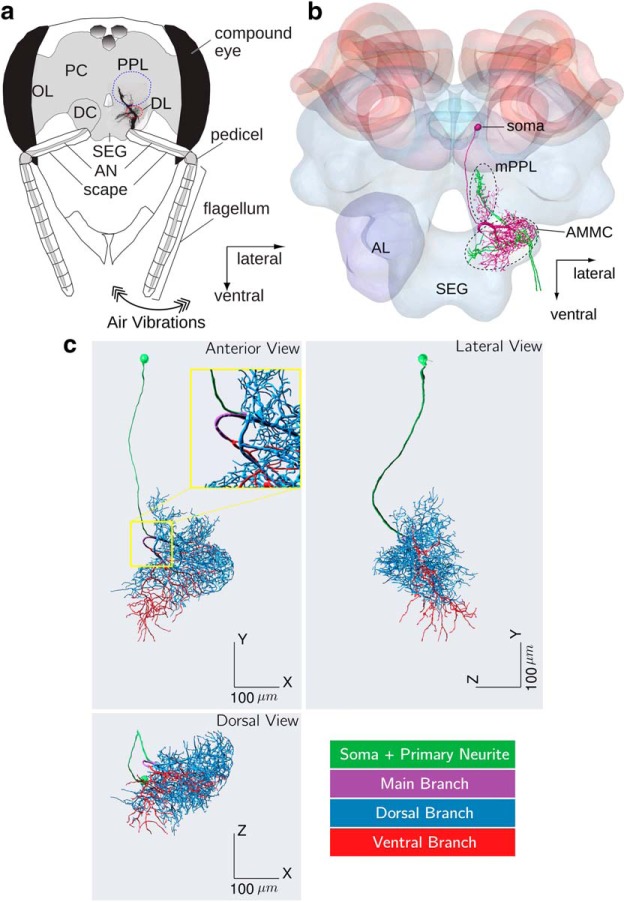
Vibration sensing, primary mechanosensory center and DL-Int-1 interneuron in the honeybee. ***a***, Airborne vibration jets produced during the waggle dance are picked up by the flagellum are transduced by sensory neurons of the JO in the pedicel and transmitted to the primary mechanosensory center of the honeybee brain, which consists of the mPPL, DL, and dSEG. Modified with permission from Ai et al. (2007), their Figure 1. ***b***, Projection patterns of sensory afferents (green) and DL-Int-1 (magenta) in the primary mechanosensory center of the honeybee brain. DL-Int-1 has dendrites running close to sensory afferents in the DL. Modified from Ai (2013), their Figure 5. ***c***, Morphology of DL-Int-1 visualized using three 2D projections. We divided DL-Int-1 morphology into four subregions for analysis. Inset, Magnified version of the region around the Main Branch. OL, Optic lobe; PC, protocerebrum; DC, deutocerebrum; AN, antennal nerve; AL, antennal lobe.

Air vibrations behind dancing honeybees are detectable only up to 15–20 mm (Michelsen, et al., 1987) and consist of low (12–25 Hz) and high (200–300 Hz) frequencies from two sources (Wenner, 1962). Vibrations produced by the wagging abdomen only contain low frequencies, whereas jets of air vibration produced by wing beats have a pulse train pattern and contain both low and high frequencies (Michelsen et al., 1987; Michelsen, 2003). Both kind of vibrations have been shown to be relevant for waggle dance communication (Michelsen et al., 1989, 1992). Honeybees can detect air vibrations using various mechanosensory organs on their bodies. Among them, the Johnston’s Organ (JO) located in the pedicel of the antennae (Fig. 1*a*) has been shown to be the primary sensory organ for detecting near-field vibrations of the waggle dance (Dreller and Kirchner, 1993). Sensory afferents of the JO project into the honeybee brain, specifically in medial posterior protocerebral lobe (mPPL) and the antennal mechanosensory and motor center (AMMC), which consists of the dorsal lobe (DL) and dorsal subesophageal ganglion (dSEG; Fig. 1*a*; Ai et al., 2007).

More than 10 groups of interneurons belonging to three categories have been identified and characterized in these regions (Ai et al., 2009, 2017; Ai, 2010, 2013) and have been shown to respond to antennal vibrations similar to those produced by air vibration jets of the waggle dance (Ai et al., 2017). In particular, a group of GABAergic interneurons called DL-Int-1 has been studied intensively and has been characterized in detail (Ai et al., 2009, 2017; Ai, 2013). However, neural responses to low-frequency vibrations produced by abdomen waggling has not yet been characterized.

DL-Int-1 somata are located in the rind of the protocerebrum and have single neurites branching and projecting to the DL, the dSEG and the mPPL, where they further branch into dense arborizations that run close to afferents (Fig. 1*b*; Ai et al., 2009). DL-Int-1 are spontaneously active and respond to vibration stimuli applied to the ipsilateral antenna. Their responses to vibration stimuli are characterized by on-phasic excitation to stimulus onset, tonic inhibition during continuous stimulation, and rebound spiking after vibration offset (Ai et al., 2009). DL-Int-1 neurons are thought to play a role in encoding the duration of the waggle phase (Ai et al., 2017), which correlates with the distance of the advertised food source from the hive (von Frisch, 1967).

As they mature, adult honeybees engage in four primary social roles—cleaners, nursers, food storers, and foragers—and perform different tasks in different roles (Seeley, 1996). Several studies have investigated the neural basis of such behavioral versatility by studying structural changes in the honeybee brain with age and social role, mainly focusing on the mushroom body (Groh, et al., 2006, 2012; Groh and Meinertzhagen, 2010). Although most developmental changes in the honeybee brain occur during pupal and larval stages (Devaud and Masson, 1999; Ganeshina et al., 2000), considerable age-dependent and experience-dependent anatomic changes have been described at the level of subregions in the adult honeybee antennal lobe (Winnington et al., 1996; Sigg et al., 1997; Morgan et al., 1998; Brown et al., 2004; Andrione et al., 2017; Arenas et al., 2013) and the mushroom body (Withers et al., 1993, 1995; Durst et al., 1994; Fahrbach et al., 1998; Wolschin et al., 2009), as well as at the level of single mushroom body neurons (Farris et al., 2001). In addition, electrophysiological properties of honeybee neurons also mature with age and experience in the antennal lobe (Wang et al., 2005) and in the mushroom body (Kiya et al., 2007). Adaptations in neural processing could be especially crucial during the transition to foraging, because, compared with in-hive activities, foraging entails several new and complex behaviors such as attending to waggle dancers, sensing the waggle dance vibration signals and decoding target location, using such information on foraging trips, and advertising newly found locations to hivemates through the waggle dance. Honeybees start following waggle dances only after 1 week after emergence (Ai et al., 2018b). Mechanosensory neurons in the JO of the antennae become more responsive to high-frequency waggle dance vibrations as honeybees mature from newly emerged adults to foragers (Tsujiuchi et al., 2007). It is unclear, however, to what extent neurons in central circuits processing waggle dance vibration signals show such adaptation. We therefore investigated morphological and electrophysiological changes of neurons in the primary mechanosensory center of the honeybee, focusing on DL-Int-1 neurons. To identify maturation-related adaptations in DL-Int-1, we analyzed and compared reconstructed morphologies and electrophysiological properties of neurons from newly emerged adult bees and foragers bees.

## Materials and Methods

### Honeybees

Honeybees (*Apis mellifera*) reared at Fukuoka University between 2012 and 2014 were used in this study. Experiments were conducted on more than 300 bees for investigating neurons in the primary mechanosensory center of the honeybee brain. Collected data included electrophysiological recordings and laser scanning microscopy images, which were stored in a database and classified into multiple neuron groups based on electrophysiological and morphological characteristics (Ai et al., 2017, their Table 1). In the current study, we used DL-Int-1 data from the database belonging to honeybees of two stages of maturation:

- **Newly emerged adults (age 1-3 d):** female honeybees shortly after emerging from their cell in the hive. Before the experiments, these bees were kept in isolated cages containing sugar solution and pollen.
- **Foragers (older than 10 d):** female honeybees returning from foraging with pollen on their hindlegs.

**Table 1.** Scalar morphometric measures showing significant differences

**Measure**	**Morphological subregion**	**Newly emerged** **(NE, *n* = 6)**	**Forager** **(*F*, *n* = 6)**	**Change in median value** **[(F-NE)/NE]**	***p***
Width (along *x*), μm	Main branch	10.4, 22.1, 26.5	25.2, 34.1, 61.4	+54.3%	0.004
Height (along *y*), μm	Dorsal branch	152, 236, 263	212, 268, 294	+13.6%	0.041
Total dendritic volume, ×10^3^(μm)^3^	Main branch	0.183, 0.409, 0.803	0.301, 0.829, 0.967	+103%	0.041
Average partition asymmetry	Whole arborization	0.597, 0.661, 0.653	0.561, 0.576, 0.636	−12.86%	0.041
Maximum centrifugal order	Ventral branch	13, 30, 43	12, 16, 20	−46.7%	0.043
Hausdorff fractal dimension	Ventral branch	1.14, 1.24, 1.35	1.11, 1.14, 1.2	−8.07%	0.041

Summary statistics of six scalar morphometric and topological parameters that show significant differences for at least one morphological subregion. The triplets in columns three and four represent minimum, median, and maximum values. *P* values were calculated using Mann–Whitney *U* test and a cutoff of 5% was used. Summary statistics for all 19 scalar measures and for all 4 subregions are provided in Extended data Table 1-1, Table 1-2, Table 1-3, and Table 1-4. Morphologies showed significant differences for a few scalar measures, with width, total dendritic volume, and maximum centrifugal order showing large changes.

### Experimental procedure

The experimental procedure for generating image stacks and electrophysiological response traces from honeybee vibration-sensitive interneurons has been presented in detail by Ai et al. (2017) and we describe it here briefly. After immobilization and head fixing using bee’s wax, the frontal surface of the honeybee brain was exposed by cutting away a small rectangular window between the compound eyes. Borosilicate glass electrodes filled at the tip with a dye were inserted into the primary mechanosensory center to record from individual neurons. Three dyes were used: Lucifer yellow CH dilithium salt (catalog number L0259, Sigma-Aldrich), Dextran tetramethylrhodamine solution (3000 molecular weight, anionic, lysine fixable; catalog number D3308, ThermoFisher Scientific), and AlexaFluor 647 hydrazide (catalog number A20502, ThermoFisher Scientific). With the electrode stably inserted into a vibration-sensitive interneuron, sinusoidal vibration stimuli of frequency 265 Hz and duration 1 s were applied to the right antenna and responses were recorded intracellularly. Electrical signals were amplified using an amplifier (MEZ8301, Nihon Kohden), filtered to remove frequencies higher than 20 kHz and recorded using Spike2 (Cambridge Electronic Design; RRID:SCR_000903) at a sampling rate of 20.833 kHz. After recording electrical activity, a hyperpolarizing current (2–5 nA for 2–10 min) was applied to inject the dye into the neuron. Thereafter, the brains were dissected out, fixed in 4% paraformaldehyde for 4 h at room temperature, and then rinsed in phosphate buffer solution, dehydrated, and cleared in methyl salicylate for subsequent observation and imaging.

The cleared specimen containing intracellularly stained neurons were viewed from the posterior side of the brain under a confocal laser-scanning microscope (LSM 510, Carl Zeiss) with a Zeiss Plan-Apochromat 25×/numerical aperture 0.8 oil lens objective (working distance, 0.57 mm). Image stacks of the AMMC and the mPPL were taken at a resolution of 0.36 μm on the imaging plane using 1-μm-thick optical sections and stitched together digitally to obtain image stacks of complete neurons.

### Morphological subregions of DL-Int-1

To refer to specific subregions of the DL-Int-1 morphology, we adopt the following definitions (Fig. 1*c*; Ai et al., 2017):

- **Soma and primary neurite (SPN):** consists of the soma and its primary neurite until bifurcation.
- **Main branch (MB):** consists of the two daughter branches of the primary neurite until they bifurcate.
- **Dorsal branch (DB):** consists of the remaining dendritic arborization originating from the dorsal end of the MB.
- **Ventral branch (VB):** consists of the remaining dendritic arborization originating from the ventral end of the MB.
- **Whole arborization (WA):** consists of the MB, the DB, and the VB.

### Reconstruction of morphologies

The reconstruction procedure has been described in detail by Ikeno et al. (2018). Briefly, image stacks with single dye-filled neurons were de-convolved to reduce image blurring and noise. Regions of each image stack containing the dendritic subtrees emerging from the dorsal and ventral daughter branches of SPNs (Ikeno et al., 2018, their Fig. 6*E*) were identified based on continuity of branching structure and dendritic thickness and were converted into custom image masks. Applying these masks, two image stacks were created that separately contained the identified dorsal and ventral subtrees of SPN. Morphologies of these subtrees were reconstructed from their image stacks by segmentation, pruning and smoothing using SIGEN software (Minemoto et al., 2009; RRID:SCR_016284) and combined to form the reconstruction of WA. WA was manually separated into MB, DB, and VB based on the first branching points on the two daughter branches of the primary neurite and stored in separate SWC files (Cannon et al., 1998) for morphometric analyses.

### Morphological comparison using scalar measures

Morphologies of DL-Int-1 neurons from newly emerged adult and forager honeybees were compared using 19 widely used metric and topological measures (Uylings and van Pelt, 2002; Scorcioni et al., 2008; Peng et al., 2014). These measures were calculated using a modified version of the BTMORPH software v2.2.1 (Torben-Nielsen 2014; RRID:SCR_003566; code: https://github.com/wachtlerlab/btmorph_v2), Vaa3d v3.447 (Peng et al., 2014; RRID:SCR_002609), and pyVaa3d (code: https://github.com/ajkswamy/pyVaa3d, v0.4). Mann–Whitney *U* test was used for calculating the significance of differences between the maturation levels with a cutoff of 5%.

### Spatial registration

Preliminary visual comparisons indicated that DL-Int-1 morphologies had differences in translation, rotation and scaling that could have resulted from structural differences between honeybee brains as well as from fixation and dehydration artifacts caused during experimentation. Therefore, we coregistered all DL-Int-1 morphologies to a common frame of reference using the Reg-MaxS-N software (Kumaraswamy et al., 2018; code https://doi.org/10.12751/g-node.feee47; RRID:SCR_016257). Reg-MaxS-N estimates and removes differences between morphologies by translation, rotation, and scaling, successively refining estimates of differences at multiple spatial resolutions. In this study we used spatial resolutions of 160, 80, 40, and 20 μm. Morphologies from newly emerged adult and forager bees were coregistered in two steps. First, newly emerged adult and forager morphologies were coregistered separately using Reg-MaxS-N (Kumaraswamy et al., 2018). Then, the two resulting groups of morphologies were brought to the same frame of reference by coregistering the unions of the points of all morphologies in a maturation group using Reg-MaxS (Kumaraswamy et al., 2018).

To control for parameter choice during spatial registration, the procedure above was repeated using multiple parameter sets. Newly emerged adult and forager morphologies were each coregistered separately using three initial references to generate three sets of registered morphologies for each maturation level. Taking all possible combinations of these sets, nine sets of all 12 morphologies were created, which were in turn registered together. All other parameters remained the same for the nine sets (for all parameters, see Extended data Fig. 2-1).

**Figure 2.**
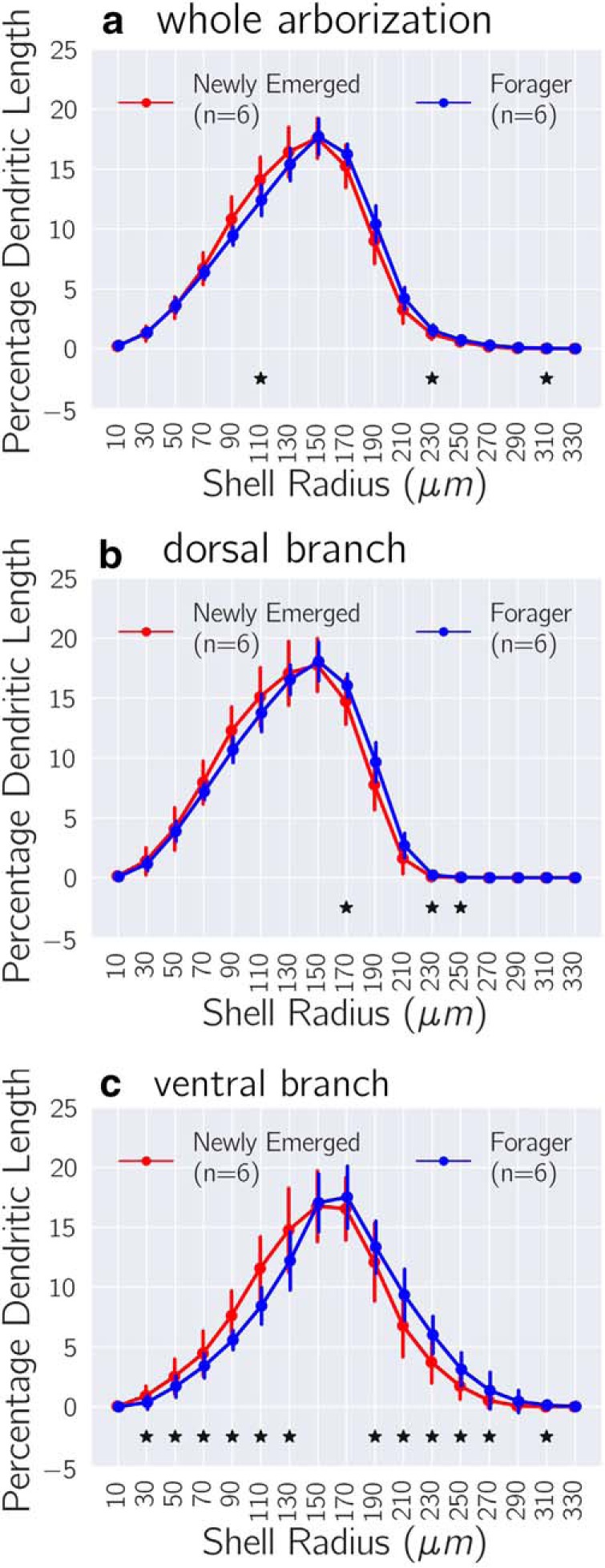
Changes in radial distribution of dendritic density. Comparison of PDL_shell_ calculated for dendrites contained in concentric spherical shells of thickness 20 μm for the WA, DB, and VB, respectively, in ***a***, ***b***, and ***c***. Solid circles indicate means and error bars indicate SD, both of which were calculated by pooling PDL_shell_ values across registration parameters (Extended data Figure 2-1). Asterisks indicate a significant difference in PDL_shell_ between maturation levels independent of registration parameters (see Extended data Figure 2-1). ART two-way ANOVA was used for factor analysis with a *p* value cutoff of 5%. These comparisons indicate a redistribution of dendritic length during maturation, with reductions in proximal parts and increases in distal parts of DL-Int-1 morphologies.

10.1523/ENEURO.0454-18.2019.f2-1 Figure 2-1 ***a***, Differing Parameters among the nine parameters sets used for registration. Among the nine parameter sets used for registration, the morphologies used as initial references for coregistering separately newly emerged adults and foragers were different and the experimental identifiers of these morphologies are listed here. Three initial references were used each for newly emerged adults and foragers and taking all possible combinations of these resulted in nine sets of parameters. ***b***, Common Parameters among the nine parameters sets used for registration. Parameters other than the initial references were common among the nine parameter sets. For parameter description, see https://web.gin.g-node.org/ajkumaraswamy/regmaxs/src/master/regmaxsn/core/RegMaxSPars.py. Download Figure 2-1, DOC file.

### Morphological comparison using spherical shells

The radial distribution of dendritic length was compared between the two maturation levels by dividing the space containing the morphologies into spherical shells of thickness 20 μm, similar to Sholl analysis (Sholl 1953; Uylings and van Pelt, 2002; Langhammer et al., 2010; Garcia-Segura and Perez-Marquez, 2014), which has been shown to be effective in analyzing morphologies (Cuntz et al., 2008; Luebke et al., 2015; O’Neill et al., 2015). As a natural extension of Sholl analysis, we used the measure dendritic length to quantify changes in dendritic arborization during maturation. For every shell, we calculated *PDL_shell_*, which is the percentage of dendritic length of a morphology contained in the shell. Using two-way ANOVA (Wobbrock et al., 2011), we tested whether, in each shell, *PDL_shell_* (1) was significantly different between newly emerged adults and foragers and (2) showed no significant dependence between the effects caused by maturation and registration parameters. The tests used a cutoff level of significance of 5% after Bonferroni correction (Bland and Altman, 1995; McDonald, 2014). This analysis was not applied to MB morphologies because most of them had no branching points and comprised of single stretches of dendrites spanning less than 50 μm.

### Morphological comparison using 3D voxels

To compare the morphologies with an even finer spatial granularity, we analyzed non-overlapping 3D voxels of size 20 μm. For each voxel, we calculated *PDL_voxel_*, which is the percentage dendritic length of a morphology contained in the voxel. Note that, because all the voxels had the same volume, changes in *PDL_voxel_* are proportional to changes in average dendritic density. The same criteria as in the previous analysis were used for identifying voxels for which *PDL_voxel_* changed significantly during maturation, independent of registration parameters. To visualize the changes in dendritic density, we calculated for each voxel the normalized change in *PDL_voxel_* as follows:

$${△PDL}_{voxel}^{norm}=\frac{\overset{¯}{PDL_{voxel}^{f}}-\overset{¯}{PDL_{voxel}^{n}}}{\overset{¯}{PDL_{voxel}}},$$

where, for a given voxel, $\overset{¯}{PDL_{voxel}^{f}}$ is the average *PDL_voxel_* for forager morphologies across registration parameters and honeybee samples, $\overset{¯}{PDL_{voxel}^{n}}$ is the average *PDL_voxel_* for newly emerged adult morphologies across registration parameters and honeybee samples; and $\overset{¯}{PDL_{voxel}}$ is the average *PDL_voxel_* across all maturation levels, registration parameters, and honeybee samples.

### Morphological comparison using proximal and distal partitions

We divided the space containing the morphologies into proximal and distal partitions based on the remoteness of morphological nodes from their roots, which was quantified using the measure %*PL*:

$$\%PL=100\times \frac{PL_{root}}{PL_{root}+PL_{term}^{\max}},$$

where *PL_root_* is the distance along the dendritic tree, also called path-length, between the node and the root; and $PL_{term}^{\max}$ is the maximum of the path-lengths between the node and all terminals in the sub-tree emanating from the node. A voxel was classified to be distal if the median value of %*PL*, calculated across maturation levels, registration parameters and honeybee samples was more than 90. The significance of differences in *PDL_voxel_* between newly emerged adults and foragers were calculated separately for proximal and distal partitions using aligned rank transform (ART) two-way ANOVA (Wobbrock et al., 2011) and the same criteria as in previous analysis.

### Analysis of electrophysiology

The physiological response of DL-Int-1 to continuous vibration stimuli applied to the antenna consisted of on-phasic excitation followed by a tonic inhibition and offset rebound (Ai et al., 2009). We defined four time periods for analyzing the electrophysiological activity of DL-Int-1 (Fig. 4*b*):

- **Spontaneous activity:** 3 s period preceding stimulus onset.
- **On-phasic response:** first 75 ms after stimulus onset.
- **Inhibitory response:** from the end of on-phasic response until stimulus offset.
- **Rebound response:** a 75 ms period after a delay of 25 ms from stimulus offset.

Raw data of electrophysiological recordings were read from Spike2 files using NEO v0.5 (Garcia et al., 2014; RRID:SCR_000634), stored using the NIX format v1.4.5 (Stoewer et al., 2014; RRID:SCR_016196) and analyzed using custom Python scripts. Trials were time-aligned to stimulus onset and time-resolved estimates of average firing rates were generated using adaptive kernel density estimation (Shimazaki and Shinomoto, 2010; Implementation: https://github.com/cooperlab/AdaptiveKDE). The distribution of spike train features such as spike rates and spike times were visualized using the “violinplot” function of the Python package seaborn (Waskom et al., 2018). This function uses kernel density estimation to estimate continuous distributions using Gaussian kernels and Scott’s formula for bandwidth calculation (Härdle et al., 2004, p 73). Mann–Whitney *U* test was used for calculating the significance of differences in response features with a cutoff of 5%.

We quantified the strength of inhibition relative to spontaneous activity by calculating Relative Inhibition, defined as follows:

$$Relative\;Inhibition=1-\frac{Firing\;Rate\;during\;Inhibitory\;Response\;period}{Firing\;Rate\;during\;Spontaneous\;Activity\;period}$$

### Computational environment, code, and data availability

Data preprocessing and analysis were conducted on a desktop computer with an 8-core Intel i7 Processor, 16 GB of RAM running Ubuntu 16.04. The data used for this study are available online on the repository GIN (https://doi.org/10.12751/g-node.e70cb4). Analysis of morphologies and electrophysiological activities was done using custom Python (RRID:SCR_008394) scripts, which are available online (https://github.com/wachtlerlab/GJEphys and https://github.com/wachtlerlab/GJMorph, respectively; Extended Data 1).

10.1523/ENEURO.0454-18.2019.ed1 Extended Data 1 This ZIP file contains all the code used to generate the results of this publication. The code is organized into two folders: (1) GJEphys-master, which contains code for analyzing and plotting electrophysiology data and (2) GJMorph-master, which contains code for analyzing morphology data. Download Extended Data 1, ZIP file.

## Results

### Data collection

Sharp electrodes were inserted into DL-Int-1 neurons in the honeybee brain to record electrophysiological activity as well as to inject dye for imaging neuron morphology. Only about 10% of electrode insertions yielded useful data because honeybee brains were not transparent enough for visually targeted electrode insertion and DL-Int-1 neurons were encountered in about one-third of such insertions (Ai et al., 2017). Furthermore, maintaining the electrode within the neuron long enough to obtain sufficient electrophysiological data were difficult especially for newly emerged adult honeybees, as their brains were soft and infirm. Our data of DL-Int-1 neurons from newly emerged adults were therefore limited to six samples with sufficient data for analysis. For the comparative analysis, we chose six forager samples from our database matching the response pattern of the neurons from newly emerged adults.

### Morphological adaptations

The four subregions of DL-Int-1 morphology—the WA, MB, DB, and VB (see Materials and Methods, Reconstruction of morphologies)—were compared separately to investigate changes during maturation.

### Analysis 1: scalar morphometrics

We first compared the morphologies using whole-cell scalar measures, which detect net overall changes in morphological subregions as they combine data from all dendrites. Table 1 lists the measures that showed significant differences between the morphologies of newly emerged adult and forager DL-Int-1 neurons for at least one subregion (for summary statistics of WA, MB, DB, and VB, see Extended data Table 1-1, Table 1-2, Table 1-3, and Table 1-4, respectively). MB and VB showed significant differences between newly emerged and forager DL-Int-1 neurons for two measures each, whereas DB and WA had one measure each with a significant difference. The changes were neither consistent across morphological subregions nor highly significant (*p*-values between 1 and 5%) and the number of measures showing significant differences were consistent with the number false-positives expected. Hence, at the level of whole-neuron morphological measures, significant changes could not be detected. However, these results did not exclude the possibility of localized changes in dendritic arborization. Therefore we investigated the morphologies at finer spatial scales.

Table 1-1 Summary statistics of 19 scalar morphometric measures applied to the whole arborization subregion of DL-Int-1 morphologies. The triplets in columns two and three represent minimum, median, and maximum values. Column four contains *p* values calculated using Mann–Whitney *U* test for differences between newly emerged adults and foragers. Measures with *p* values <5% are highlighted in red. Download Table 1-1, DOC file.

Table 1-2 Summary statistics of 19 scalar morphometric measures applied to the main branch subregion of DL-Int-1 morphologies. The triplets in columns two and three represent minimum, median, and maximum values. Column four contains *p* values calculated using Mann–Whitney *U* test for differences between newly emerged adults and foragers. Measures with p values <5% are highlighted in red. It was not possible to calculate statistics for some measures (marked N/A) as one or more morphologies of newly emerged adult or forager DL-Int-1 neurons had no bifurcations in the main branch. Download Table 1-2, DOC file.

Table 1-3 Summary statistics of 19 scalar morphometric measures applied to the dorsal branch subregion of DL-Int-1 morphologies. The triplets in columns two and three represent minimum, median, and maximum values. Column four contains *p* values calculated using Mann–Whitney *U* test for differences between newly emerged adults and foragers. Measures with *p* values <5% are highlighted in red. Download Table 1-3, DOC file.

Table 1-4 Summary statistics of 19 scalar morphometric measures applied to the ventral branch subregion of DL-Int-1 morphologies. The triplets in columns two and three represent minimum, median, and maximum values. Column four contains *p* values calculated using Mann–Whitney *U* test for differences between newly emerged adults and foragers. Measures with *p* values <5% are highlighted in red. Download Table 1-4, DOC file.

### Analysis 2: radial distribution of dendritic length

Before detailed spatial analysis, DL-Int-1 morphologies of newly emerged adults and foragers were coregistered to a common frame of reference (see Materials and Methods, Spatial registration) to establish spatial correspondence. Figure 2 compares the radial distributions of *PDL_shell_* between newly emerged adults and foragers for WA, DB, and VB and highlights those spherical shells for which *PDL_shell_* changed significantly during maturation, independent of registration parameters (see Materials and Methods, Morphological comparison using spherical shells). WA showed reductions in *PDL_shell_* at 110 μm, whereas DB showed an increase at 170 μm during maturation. VB showed reductions up to 130 μm and increases for between 190 and 270 μm during maturation. These comparisons support a consistent redistribution of *PDL_shell_* over shells, with reductions in proximal regions and increases in distal regions of the morphology.

To exclude that the observed pattern of proximal reduction and distal increase in dendritic density was a result of a residual scaling difference because of incomplete convergence of the iterative registration process, we repeated the coregistration of the morphologies with different starting conditions, using versions of the newly emerged adult morphologies that were artificially scaled up by 10 or 15%. In both cases, the results were the same as without the scaling (data not shown), confirming that the observed differences in the spatial distributions of dendritic length were not caused by scaling differences.

### Analysis 3: local dendritic length

**Figure 3.**
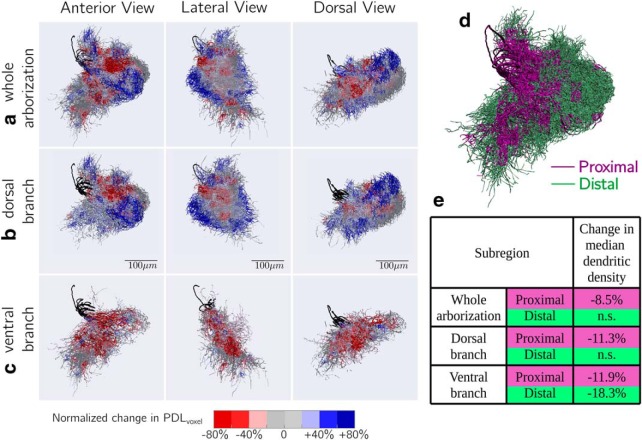
Region-dependent changes in dendritic density. All 12 DL-Int-1 morphologies visualized together after co-alignment, highlighting regions that show significant differences in PDL_voxel_ during maturation. ***a***, WA, ***b***, DB, and ***c***, VB. A voxel was highlighted if ART two-way ANOVA indicated that maturation had a significant effect on PDL_voxel_ independent of registration parameters. The dendrites were colored with normalized change in PDL_voxel_ (see Materials and Methods, Morphological comparison using 3D voxels; for distributions, see Extended data Figure 3-1). The MBs are colored in black. ***d***, The space containing the morphologies was divided into proximal and distal partitions based on distances along the dendritic tree of a node from the root and terminals in its subtree (for detailed 3D view for all subregions, see Extended data Figure 3-2). ***e***, Changes in median PDL_voxel_ in proximal and distal partitions of each subregion. “n.s.” indicates that maturation did not have a significant effect on PDL_voxel_ independent of registration parameters when tested with ART two-way ANOVA (for distributions, see Extended data Figure 3-3).

To investigate the observed changes in the radial distribution of dendritic length at finer spatial detail, we compared the morphologies at the scale of voxels of size 20 μm using *PDL_voxel_* (see Materials and Methods, Morphological comparison using 3D voxels). Figure 3*a*–*c* visualizes the magnitude and spatial distribution of normalized change in *PDL_voxel_* (see Materials and Methods, Morphological comparison using 3D voxels; Extended data Fig. 3-1) for voxels showing significant changes in the WA, DB, and VB using a color map (see Extended data Figure 3-1 for distributions). Consistent with indications from the previous analysis, some proximal voxels showed reductions in *PDL_voxel_*, whereas some distal voxels showed increases. To quantify the observed changes more concretely, we divided the space containing the morphologies into proximal and distal partitions (Fig. 3*d*, Extended data Figure 3-2; see Materials and Methods, Morphological comparison using proximal and distal partitions). Pooling values across voxels in each partition, we used two-way ANOVA to test for significant changes in *PDL_voxel_* during maturation independent of registration parameters (Fig. 3*e*, Extended data Figure 3-3, see Materials and Methods, Morphological comparison using proximal and distal partitions). WA, DB, and VB showed significant reductions of 8.5, 11.3, and 11.9%, respectively, in median *PDL_voxel_* for the proximal partition. Whereas WA and DB did not show a significant change in median *PDL_voxel_* for the distal partition, VB showed a significant reduction of 18.3%. Thus, there was a region-dependent reduction in the dendritic density of DL-Int-1 with more subregions showing a reduction for proximal parts of the arborization than for distal parts.

10.1523/ENEURO.0454-18.2019.f3-1 Figure 3-1 Distributions of normalized change in PDL_voxel_ for WA, DB, and VB. The distributions were calculated by smoothing histograms using Gaussian filters with standard deviation equal to 0.001 times the SD of the data. The box plots indicate quartile deviations. The distribution for the DB had more positive values, with values as high as 119%, whereas the distribution for the VB had more negative values with values as low as −15%. Both distributions had several points lesser than the first quartile and greater than the third quartile. Download Figure 3-1, EPS file.

10.1523/ENEURO.0454-18.2019.f3-2 Figure 3-2 Proximal and distal partitions. All 12 DL-Int-1 morphologies visualized together after co-alignment, illustrating proximal and distal partitions. ***a***, WA, (***b***) DB, and (***c***) VB. The space containing the morphologies was divided into proximal and distal partitions based on the path lengths along the dendritic tree between a morphological node and the root as well as the morphological node and its downstream terminals (see Materials and Methods). Download Figure 3-2, EPS file.

10.1523/ENEURO.0454-18.2019.f3-3 Figure 3-3 Distributions of P DL voxel restricted to proximal and distal partitions. The distributions were calculated by smoothing histograms using Gaussian filters with SD equal to 0.001 times the SD of the data. The distributions were normalized to have equal areas. Horizontal white markers indicate mean values of the distributions. An asterisk indicates a significant difference in *p* DL voxel between maturation levels independent of registration parameters. ART two-way ANOVA was used for factor analysis with a *p* value threshold of 5%. Although all subregions showed significant difference for the proximal partitions, only VB showed significant difference for the distal partition. Download Figure 3-3, EPS file.

### Electrophysiological adaptations

**Figure 4.**
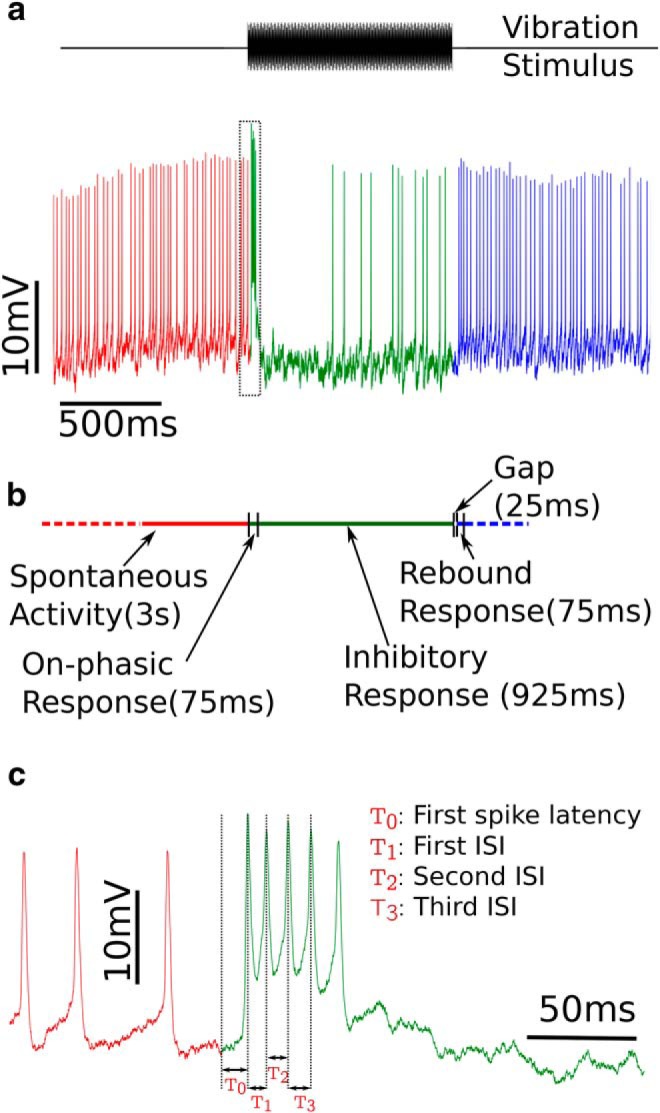
Definition of activity periods and spike timing features of electrophysiological responses. ***a***, An example response of DL-Int-1 to 1 s long vibration stimulus of 265 Hz. Activity before stimulus is colored in red, activity during stimulus in green and activity after stimulus in blue. ***b***, The definitions of the four activity periods used for analyzing electrophysiological properties of DL-Int-1. ***c***, The trace contained in the dotted rectangle in ***a*** is magnified and the four spike timing features, T_0_, T_1_, T_2,_ and T_3_ are defined on it.

**Figure 5.**
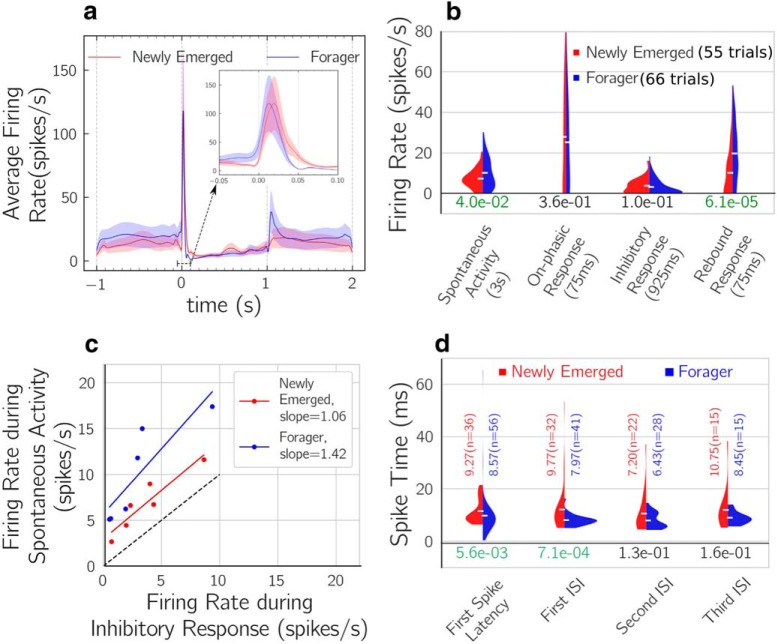
Analysis of electrophysiological properties. ***a***, Comparison of the average firing rate profiles of newly emerged adult and forager DL-Int-1 neurons. Smoothed estimates of time-resolved average firing rates were calculated from responses aligned to stimulus onset using adaptive kernel density estimation (Shimazaki and Shinomoto, 2010). Solid lines indicate average firing rates, whereas shaded regions indicate 95% confidence intervals. Inset, Average firing rate during on-phasic response with expanded time scale. ***b***, Comparison of firing rates during four activity periods. The filled areas represent firing rate distributions estimated using kernel density estimation (see Methods, Analysis of electrophysiology). The distributions were normalized to have equal areas. Horizontal white markers indicate mean values of the distributions. The numbers below the distributions are *p* values calculated using Mann–Whitney *U* test. *P*-values <5% are highlighted in green. Firing rates during spontaneous activity and rebound response showed significant increases. ***c***, Comparison of the strength of inhibition relative to spontaneous activity by plotting the firing rates during the two periods against each other. Lines were fit using linear least-squares regression. The dashed line indicates the line of slope 1. ***d***, Comparison of the timing of the first four spikes of the response using first spike latency, first ISI, second ISI, and third ISI. The distributions were estimated using Kernel Density estimation and normalized to have the same area. The numbers under the distributions are *p* values calculated using Mann–Whitney *U* test. Horizontal white markers indicate mean values, which are also shown above the distributions with sample numbers in parentheses. Spiking response was faster in foragers compared with newly emerged adults with significant reductions in first spike latency and first ISI.

Comparison of time-resolved firing rate estimates of the responses of DL-Int-1 neurons (see Fig. 5*a*) indicated increased spontaneous activity and a remarkable increase in firing rate just after stimulus offset in foragers compared with newly emerged adults. These observations were quantified by comparing the firing rates during the four activity periods (Fig. 4*b*) as well as the spike timing during on-phasic response (Fig. 4*c*). Figure 5*b* summarizes the comparison of firing rates for the four periods. Average spontaneous firing rate showed a significant increase of 39.6%. Average firing rates during on-phasic and inhibitory response periods did not show significant changes, but average firing rate during rebound response nearly doubled, increasing by 94.75%. Thus, DL-Int-1 responses showed stronger spontaneous activity and rebound response.

DL-Int-1 is GABAergic and likely part of a disinhibitory network (Ai et al., 2017; Kumaraswamy et al., 2017). Therefore, increased spontaneous rate and poststimulus rebound in foragers compared with newly emerged adults is expected to result in enhanced strength of the inhibitory signal indicating antennal vibration. To quantify the signal strength of inhibition in DL-Int-1 relative to the level of spontaneous activity, we calculated Relative Inhibition (see Materials and Methods, Analysis of electrophysiology) by plotting the firing rates during the two response periods against each other (Fig. 5*c*). In general, higher spontaneous spiking was associated with higher spike rates during inhibition in DL-Int-1 neurons, but the difference in firing rates between spontaneous activity and inhibitory response was larger in foragers than in newly emerged adults. Quantitatively, Relative Inhibition was 0.42 ± 0.24 in newly emerged adults, but 0.66 ± 0.18 in foragers (mean ± SE; *p*-value: 1.34%, Welch’s unequal variance *t* test). Additionally, least-squares regression in Figure 5*c* indicated that the difference became larger with firing rate level in foragers (slope of 1.42 for foragers vs 1.06 for newly emerged adult), indicative of faster response dynamics in foragers compared with newly emerged adults.

In addition to changes in activity levels during different periods, comparison of firing rate profiles during on-phasic response (Fig. 5*a*, inset) indicated a change in the timing of the excitation peak. We investigated this by comparing the first spike latency, first interspike interval (ISI), second ISI, and third ISI during on-phasic response (Fig. 5*d*). All four spike-timing features showed a systematic reduction during maturation, with average values of first spike latency and first ISI showing significant reductions of 1.76 and 4.01 ms, respectively. Thus, spike timing of DL-Int-1 neurons during on-phasic excitation showed a systematic reduction indicative of response speed-up.

## Discussion

In this study, we have compared morphological and physiological properties of an identified vibration-sensitive interneuron, DL-Int-1, between newly emerged adult and forager honeybees. Although comparisons of whole-cell scalar morphometric measures showed no major differences in broad dendritic structure and gross morphological features, detailed spatial analyses revealed region-dependent reduction in dendritic density with stronger reductions in proximal parts than in distal parts. This is consistent with findings from previous studies that investigated changes during maturation in the honeybee antennal lobe (Devaud and Masson, 1999) and mushroom body (Farris et al., 2004), which concluded that most of the process of dendritic maturation is completed before emergence, although minor age-dependent and age-independent changes continue for the first few weeks. Such region-dependent changes have also been shown in dendritic arborizations of Kenyon cells in the adult honeybee (Farris et al., 2001) as well as in the paper wasp (Jones et al., 2009). Comparison of the electrophysiological responses of DL-Int-1 to vibration stimuli between newly emerged adult and forager honeybees showed increased spontaneous activity and stronger poststimulus rebound during maturation, whereas the qualitative pattern of response remained unchanged. Similar response enhancements have been reported for odor representation in honeybees (Wang et al., 2005), where odor-dependent activity patterns in antennal lobe glomeruli were similar in newly emerged adult and forager honeybees, with older neurons showing higher spiking rates and more active glomeruli.

### Effect on network connectivity

Although the DB and VB subregions of DL-Int-1 showed reductions in dendritic density during maturation in the proximal parts, only VB showed reduction in dendritic density in the distal parts. The observed changes thus indicate region-dependent pruning in distal parts. DB and VB arborize in different sets of brain neuropils; the DB arborizes in the mPPL and the DL, whereas the VB arborizes in the DL and the dSEG. DB and VB could therefore be the areas where DL-Int-1 connects to different networks and the observed changes in morphology during maturation might reflect a refinement of the network connectivity.

The AMMC region of the honeybee brain is a center for multisensory integration, especially for waggle dance signals produced by wing beats (Ai and Hagio, 2013; Brockmann and Robinson, 2007). DL-Int-1 arborizes with fine terminals and boutons in the AMMC (Ai et al., 2009), indicating the presence of synaptic inputs and outputs in the region (Petralia et al., 2016). The observed decreases in dendritic density could be associated with changes in the synaptic ultra-structure, similar to that shown in the honeybee mushroom body (Groh et al., 2012; Muenz et al., 2015). However, more studies with synaptic labeling and higher resolution imaging are required to clarify such changes.

### Enhanced inhibition

The observed changes in electrophysiological activity of DL-Int-1 could reflect an enhancement of features relevant for network processing of high-frequency components of waggle dance vibration signals in the honeybee primary mechanosensory center. Spontaneous firing rates were significantly higher in foragers than in newly emerged adults, while firing rates during inhibitory responses were similar. Thus, the inhibitory response to vibration stimuli was relatively stronger in foragers than in newly emerged adults. Because DL-Int-1 is itself inhibitory and possibly part of a disinhibitory network processing waggle dance air vibration signals (Ai et al., 2017, 2018a; Kumaraswamy et al., 2017), the observed strengthening of relative inhibition could result in more effective disinhibition. Furthermore, the strength of post-inhibitory rebound doubled during maturation. Because inhibition coupled with post-inhibitory rebound has been suggested to play an important role in processing temporal signals in insects (Ai et al., 2018a) and specifically in detecting temporal features (Hedwig 2016; Alluri et al., 2016; Naud et al., 2015; Yamada et al., 2018), our results suggest improved detection of information encoded in the temporal features of waggle dance air vibration signals in forager honeybees.

### Effects of morphological changes on physiology

The observed changes in neuron morphology of DL-Int-1 are consistent with a refinement process during maturation that may lead to improved propagation and processing of vibration signals in foragers compared with newly emerged adults. The broad structure of the DL-Int-1 did not show major changes during maturation, with the dendritic branches of the DB and VB extending to similar regions in the honeybee brain. Under this condition and assuming unchanged membrane parameters, reduced dendritic length in proximal regions could indicate lower electrical resistance, and thus, for passive propagation, lower signal attenuation through the DB and VB (Rall and Rinzel, 1973; Ferrante et al., 2013). However, clarification of these effects using multi-compartmental neuron simulations is currently limited by the lack of data about membrane parameters. The potential for such simulations is nonetheless high because morphological reconstructions for several newly emerged adult and forager DL-Int-1 neurons are available.

### Changes in response properties: neuron or network?

The response of DL-Int-1 neurons to vibration stimuli applied to the antennae is the combined effect of its inputs and its own intrinsic electrophysiological properties (Ai et al., 2017). In this study, significant increases were seen in spontaneous activity and the strength of inhibition relative to spontaneous activity, as well as in the strength of post-inhibitory rebound. These changes are likely because of maturation of the electrophysiological properties of DL-Int-1 as well as its connected neuronal network. Specifically, the adaptations in the strength of inhibition relative to spontaneous activity could have a stronger dependence on network factors as DL-Int-1 is inhibitory and is believed to be inhibited in turn as part of a disinhibitory network in the honeybee primary mechanosensory center (Ai et al., 2017; Kumaraswamy et al., 2017). Further, the JO sensory neurons, which transduce antennal vibrations of the waggle dance and project close to the dendrites of DL-Int-1, show stronger responses to antennal deflection in foragers than in newly emerged adults (Tsujiuchi et al., 2007). This adaptation in the responses of JO neurons can also play a role in shaping the response properties of DL-Int-1 during maturation. Clarification of the contribution of these sources would be beneficial for further understanding the role of DL-Int-1 in networks that process air vibration jets of the waggle dance in the primary mechanosensory center of the honeybee.

### Genetically programmed aging or foraging experience?

In this study we have quantified morphological and physiological changes in DL-Int-1 as honeybees mature from newly emerged adult bees (1–3 d old) to forager bees (>10 d old). There are two major factors that could cause such changes during maturation: genetically programmed aging and foraging experience. Further studies with age-controlled older honeybees with no foraging experience are required to elucidate the effect of these factors on DL-Int-1 maturation.

### Linking observed changes to behavior

After successful foraging, honeybees return to their hive and perform the waggle dance, during which they produce patterns of air vibration pulses. Follower bees detect these pulses and gain information about the distance and direction of the advertised food sources (Michelsen et al., 1992; Landgraf, 2013). It has been argued that DL-Int-1 plays a role in the networks encoding information from air vibration jets of the waggle dance into neural signals (Ai et al., 2017; Kumaraswamy et al., 2017). The observed changes in DL-Int-1 suggest that neurons and networks processing waggle dance communication signals undergo functional and structural refinement as the honeybee matures that could prepare the bees to better process those important signals as foragers.

## Synthesis

Reviewing Editor: Elizabeth Cropper, Mt Sinai Sch Med Decisions are customarily a result of the Reviewing Editor and the peer reviewers coming together and discussing their recommendations until a consensus is reached. When revisions are invited, a fact-based synthesis statement explaining their decision and outlining what is needed to prepare a revision will be listed below. The following reviewer(s) agreed to reveal their identity: NONE.

This is a resubmission and all the comments from the previous review have been adequately addressed.
