# Supplementary material for: Adaptations during Maturation in an Identified Honeybee Interneuron Responsive to Waggle Dance Vibration Signals
Source: eNeuro. 2019 Sep 5;6(5):ENEURO.0454-18.2019. doi: 10.1523/ENEURO.0454-18.2019 (PMC6731536; doi:10.1523/ENEURO.0454-18.2019)
Supplement: Figure 2-1 — a, Differing Parameters among the nine parameters sets used for registration. Among the nine parameter sets used for registration, the morphologies used as initial references for coregistering separately newly emerged adults and foragers were different and the experimental identifiers of these morphologies are listed here. Three initial references were used each for newly emerged adults and foragers and taking all possible combinations of these resulted in nine sets of parameters. b, Common Parameters among the nine parameters sets used for registration. Parameters other than the initial references were common among the nine parameter sets. For parameter description, see https://web.gin.g-node.org/ajkumaraswamy/regmaxs/src/master/regmaxsn/core/RegMaxSPars.py. Download Figure 2-1, DOC file. [file sup_enu-eN-NWR-0454-18-s10.doc]

**(a)**

| **Parameter**  **Set Number** | **Initial references** | |
| --- | --- | --- |
|  | **Newly Emerged Adult** | **Foragers** |
| 1 | HB130605-1 | HB130313-4 |
| 2 | HB130605-2 | HB130313-4 |
| 3 | HB130523-3 | HB130313-4 |
| 4 | HB130605-1 | HB130322-1 |
| 5 | HB130605-2 | HB130322-1 |
| 6 | HB130523-3 | HB130322-1 |
| 7 | HB130605-1 | HB130425-1 |
| 8 | HB130605-2 | HB130425-1 |
| 9 | HB130523-3 | HB130425-1 |

**(b)**

| ****Parameter**** | ****Value**** |
| --- | --- |
| gridSizes | [160, 80, 40, 20] μm |
| transBounds | [-30, 30] μm for each of X, Y and Z axes |
| transMinRes | 1 μm |
| rotBounds | [-30, 30] degrees about each of X, Y and Z axes |
| rotMinRes | 1 degree |
| scaleBounds | [0.5, 2] for each of X, Y and Z axes |
| minScaleStepSize | 1.005 |
