# Supplementary material for: Adaptations during Maturation in an Identified Honeybee Interneuron Responsive to Waggle Dance Vibration Signals
Source: eNeuro. 2019 Sep 5;6(5):ENEURO.0454-18.2019. doi: 10.1523/ENEURO.0454-18.2019 (PMC6731536; doi:10.1523/ENEURO.0454-18.2019)
Supplement: Extended Data 1 — This ZIP file contains all the code used to generate the results of this publication. The code is organized into two folders: (1) GJEphys-master, which contains code for analyzing and plotting electrophysiology data and (2) GJMorph-master, which contains code for analyzing morphology data. Download Extended Data 1, ZIP file. [file sup_enu-eN-NWR-0454-18-s02.zip › GJEphys-master/doc/Description_Usage.docx]

Data processing pipeline for the electrophysiology data of GinJang Project

# Summary

This document contains brief description of the electrophysiological data of the Ginjang project along with brief descriptions about its generation and pipeline for processing. It also contains a step-by-step guide for using the pipeline for processing data as well as descriptions of a number of use cases of the pipeline.

# Description of Pipeline

This section contains brief descriptions of the experimental data used by the pipeline, including electrophysiology recordings and experimental metadata, followed by a description of the three main parts of the pipeline.

## **Input Data**

### Description of Experiments

Experimental data from Honeybees were collected at Fukuoka University, Japan primarily by Dr. Kazuki Kai between 2012-2014. The data was acquired to identify and characterize neurons arborizing in the honeybee dorsal lobe that respond to vibration stimuli applied to the ipsilateral antenna. A prominent subgoal of data collection was the investigation of morphological and physiological changes in DL-Int-1 with age. For this purpose, DL-Int-1 data was collected from newly emerged adult as well as forager honeybees.

The experimental procedure for generating LSM image stacks and electrophysiological response traces has been described in detail in Ai2017, doi: [10.1523/JNEUROSCI.0044-17.2017](https://doi.org/10.1523/JNEUROSCI.0044-17.2017) and I present it here briefly. Honeybees were collected from the hive and left in ice overnight to immobilze them. After fixing their head in a clamp, the frontal surface of the brain was exposed by cutting away a small rectangular window between compound eyes under a microscope. Borosilicate glass electrodes filled at the tip with a dye (Lucifer Yellow, Alexa Fluor 647 or Rhodamine) were inserted into DL-dSEG and medPPL to record from individual neurons. Different vibration simuli were applied to the right antenna, including continuous stimuli and pulse trains, and responses were recorded intracellularly. Electrical signals were amplified using an amplifier (MEZ8301, Nihon Kohden) and recorded using Spike2 (Cambridge Electronic Design; RRID: SCR_000903). After recording electrical activity, the experimenter applied a hyperpolarizing current (2–5 nA for 2–10 min) to fill the neurons with the dye. Thereafter, the brains were dissected out, fixed in 4% paraformaldehyde for 4 hours at room temperature, then rinsed in phosphate buffer solution, dehydrated, and cleared in methyl salicylate for subsequent observation and imaging.

### Experimental Time Series Data


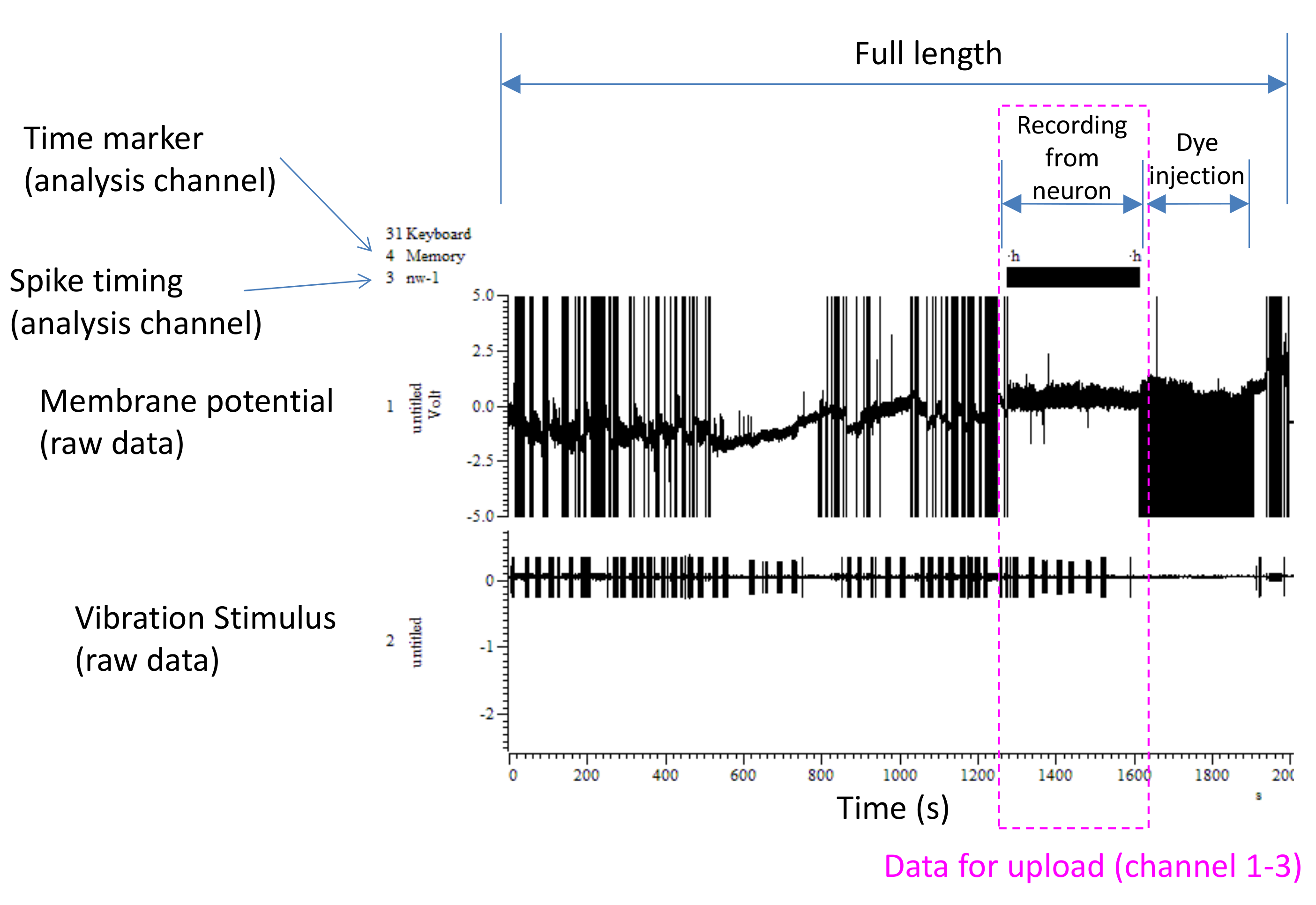

Figure 1: View of an experimental recording along with descriptions. Courtesy: Kazuki Kai.

An example of the traces recorded during an experiment is shown in Fig. 1. Each experiment consists of three periods and different actions were taken during different periods leading to different patterns on the recorded traces.

Each experiment started with a “Search Period” during which the experimenter continually applied 1-s long vibration pulses of 265Hz to check the response of the impaled cell. The experimenter also applied short mechanical vibrations to the electrode to pierce through tissue, while at the same time moving it deeper into the honeybee brain in the search of vibration sensitive cells. These mechanical electrode vibrations are seen as large-amplitude short-lived oscillations in the recorded membrane potential (Fig. 1). The Search Period continued until a vibration sensitive neuron was found, after which the electrode position was fixed. This marked the beginning of the “Recording Period”, the start and end of which was noted down as metadata.

The Recording Period was most important interval of the experiment and produces data that was most useful during analyses. Different stimulus patterns were applied during this period and the cell responses were recorded. Mainly two types of stimulus patterns were applied. The first kind was sinusoidal in nature and lasted for one second without break. Frequency was the main parameter of this kind of stimulus and all frequencies used in an experiment was noted down as metadata. Most commonly frequencies were 100, 200, 265, 300 and 400Hz. The second kind of stimulus consisted of several consecutive short sinusoidal pulses, each having the same frequency and duration. Each pair of consecutive pulses were separated by the same time interval. All sinusoidal pulses had a frequency of 265Hz. The two parameters of this kind of stimulus were pulse duration and pulse interval, and all combinations of the two parameters used in an experiment were written down as metadata . In a small number of experiments, short current pulses were applied without vibration stimulus and the responses to these current pulses can be used to estimate input resistance and membrane time constant of the cell.

The Recording Period was followed by the “Dye Injection Period” during which short current pulses were applied to inject the dye contained in the tip of the electrode into the impaled cell.

Intracellularly recorded traces were stored in SMR files using Spike2 software (Cambridge Electronic Design; RRID: SCR_000903). These files contain at least three containers for time series data called analog channels:

1. Channel 1: Trace of the membrane Potential
2. Channel 2: Trace of the vibration stimulus input
3. Channel 3 (optional): Trace of the injected current

During the experiment, the experimenter could have changed the gain of the amplifier through which membrane potential is recorded. This lead to steep jumps in membrane potential values due to change in the scale of the recorded membrane potential. The time intervals during which the gains were changed as well as the gains during those periods were recorded as metadata in the column “calibration mV/V”.

### Experimental Metadata

Experimental metadata such as experimenter name, date of experiment, stimulus applied, calibration setting and other observations during the experiment were initially written down by Kazuki Kai in a laboratory notebook and then transferred into an excel file. The description of the different metadata recorded has been provided by Kazuki Kai and available on the GinJang Owncloud at <https://filebox.g-node.org/owncloud/index.php/apps/files/?dir=%2FGinJang_data%2FGinJang_BigRawData%2FMETADATA%2FData_description_for_Neuron_Database.xlsx>. Experiments were classified by Dr. Hiroyuki Ai into categories based on the electrophysiological response properties as well as the arborization pattern of the recorded neuron. The extensive list of Experiment IDs and their metadata, along with their categorization is contained in an excel file called “neuron_database.xlsx”. These files as provided by Kazuki Kai and Hiroyuki Ai is available on GinJang Owncloud at [https://filebox.g-node.org/owncloud/index.php/apps/files/?dir=%2FGinJang_data%2FGinJang_BigRawData%2FMETADATA](https://filebox.g-node.org/owncloud/index.php/apps/files/?dir=%2FGinJang_data%2FGinJang_BigRawData%2FMETADATA%2FData_description_for_Neuron_Database.xlsx).

Since there were inconsistencies in the experimental metadata, Goekberk Alagoez and I added corrections to it. The recording periods and voltage calibration of some experiments needed corrections while voltage calibrations of other experiments were missing. We added a new metadata column to the excel file called “Intervals to exclude” where we noted down periods which high noise or large artifacts. We started with a version named, “neuron_database_20150720ha_ver6.xlsx” and the entire history of corrections can be found in GIN repository “Ginjang-Metadata” (<https://web.gin.g-node.org/ajkumaraswamy/Ginjang-Metadata>), each commit being a change to the metadata of one experiment along with comments. This should come in handy if any of these corrections need to revisited.

## Parts of Data Processing pipeline

The pipeline for processing electrophysiological data consists of three parts which were executed one after another. Each part was implemented as a class/set of functions and was executed using designated scripts. The details of the parts follow.

### Part 1: Importing Data

This part was implemented in the file “GJEphys/rawDataImport.py”. This part was executed for multiple neurons and neuron categories using the file “scripts/usedInPubs/rawDataImporter.py”. The set of actions that constitute this part of the pipeline are listed below.


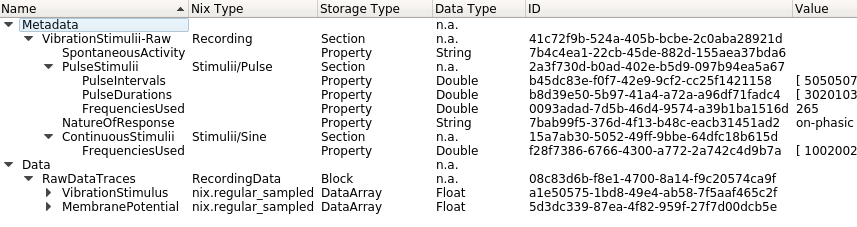

Figure 2: Overview of an NIX file after running part 1 of the pipeline to import the data of an experiment.

1. The metadata file is read in and parsed. For each experiment, the availability of a corresponding SMR file is checked.
2. Metadata values are extracted from string entries of the input metadata excel file.
3. A check is conducted if sufficient calibration information is available for each experiment.
4. The calibration information is parsed from strings. Time series data of Membrane potential, vibration stimulus and (if present) input current are read from the SMR file and trimmed to only include data with the Recording Period. These trimmed traces are calibrated by multiplying by the amplifier gains parsed from metadata.
5. A separate NIX File is created for each experiment and the metadata and data are organized as shown in Fig. 2.
6. If there is an error with importing an SMR file, the error is caught and printed to STDOUT.
7. After working through all SMRs, excel file with two columns containing information about the experiment IDs (strings) and success/failure (boolean value) is written.

### Part 2: Processing Data to identify stimuli parameters and organize them

This part is implemented in the file “GJEphys/rawDataProcess.py”. This part can be executed for multiple neurons and neuron categories using the file “scripts/usedInPubs/rawDataProcesser.py”. The set of actions that constitute this part of the pipeline are listed below.

- 1. For each experiment, the imported Nix file is read in. When this part of the pipeline successfully finishes, it would have added a section called “VibrationStimulii-Processed”. The program checks if such a section exists to determine if this NIX file has already been through this part of processing. If so, all data and metadata that would have been previously added by this part of the pipleline is removed.
  2. Spikes are detected on the membrane potential signal. Here is the procedure:
     1. apply Butterworth Filter to membrane potential trace with a cut-off frequency of 100Hz, a transition width of 40Hz and a allowed ripple of 20DB
     2. Median Value of the resulting signal is subtracted from itself.
     3. The Median Absolute deviation value of the resulting signal is calculated and used as a threshold for detecting spikes.
     4. All local maxima which satisfy the following two criteria are identified as potential spikes.
        1. It must be higher than the threshold
        2. It’s bandwidth at the threshold must fall in [0.5, 5]ms.
     5. The distribution of heights of identified potential spikes are modeled using a 5-component Gaussian Mixture Model. The intersections between consecutive Gaussian components are calculated. That intersection is chosen as spike threshold, for which separation between the means of the two Gaussian components on either side of it is maximum among all intersections.


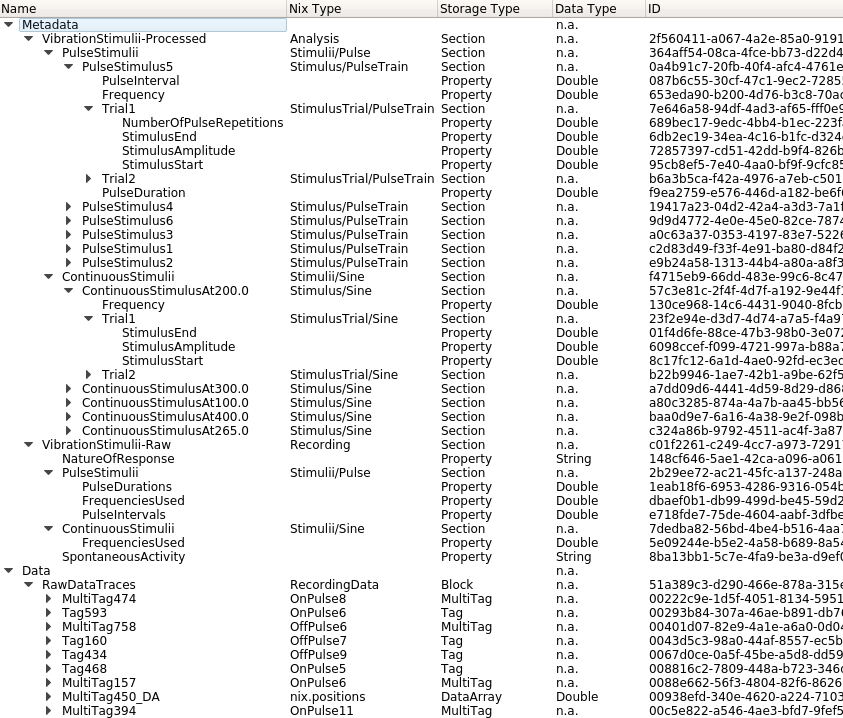

Figure 3: Overview of a NIX File after running part 2 of the pipeline to identify and organize stimuli.

- - 1. All potential spikes with height higher than the chosen spike threshold are identified as spikes.
  1. The vibration stimulus signal is down sampled and the beginning and ending of each pulse on the stimulus signal are identified.
  2. Tags are added to the traces that indicate stimulus intervals. Multitags are added to the traces that indicate spike trains associated with each pulse of the stimulus.
  3. The pulses identified on the vibration stimulus signal are sorted into continuous stimuli pulse train stimuli based on length of the pulse interval, the length of the gap to the preceding pulse and the length of the gap to the succeeding pulse.
  4. Additional metadata are written, in the form of the section “VibrationStimulii-Processed”. See Fig. 3 for details.
  5. The Nix File is written to the disk.
  6. When the processing procedure fails with an error for an experiment, the error is caught and its message is printed to STDOUT and the next experiment is processed. Success/Failure of experiments are saved in an excel file.

### Part 3: Fetching response traces and spike trains

This part is implemented in the file “GJEphys/rawDataAnalyse.py”. It contains the class “RawDataAnalyser” the following convenience methods for fetching response traces, spike trains and other information from processed NIX Files:

- 1. **getContStats:** Given the path of a processed NIX File, this function returns a dictionary. The frequencies used for continuous stimuli are the keys and lists of strings are values, where each string is formatted as “TrialX” where X is replaced by the trial number. This essentially contains an overview of the different parameters and number of trials per parameter used for continuous stimulus.
  2. **getPulseStats:** Given the path of a processed NIX File, this function returns tabular data in the form of a pandas (https://pandas.pydata.org/) DataFrame. The table contains data pertaining to one trial in each row organized in the following columns:
     1. Pulse Duration (ms)
     2. Pulse Interval (ms)
     3. Trial Label
     4. Frequency (Hz)
     5. Number of Pulse Repetitions
     6. Time of Start of Pulse Stimulus (s)

This data provides an overview of the parameter combinations and their associated trial repetitions for pulse train stimulus.

- 1. **getContResps:** Given a processed NIX File, this function retrieves the responses to all continuous stimuli and returns them organized in nested dictionaries. The first level of nesting has frequency of the stimulus as keys. The second level of nesting has the trial number (starting from 0) as keys. The third level of nesting has at max three keys and their associated response traces as neo (http://neuralensemble.org/neo/) analogsignals. The possible keys are “BeforeStimulus”, which corresponds to an interval of 3s before stimulus onset, “DuringStimulus”, the interval between stimulus onset and offset and “AfterStimulus”, an interval of 3s after stimulus offset.

Note that this function has input arguments for filtering the set of responses retrieved. The argument “freqs” can be used to filter responses based on stimulus frequency and “types” can used to specify the time intervals among “BeforeStimulus” , “DuringStimulus”, “AfterStimulus” that are fetched. Not specifying any arguments will fetch responses for all time intervals and frequencies.

- 1. **getContSpikes:** Given a processed NIX File, the function retrieves the spike times detected during responses to continuous stimuli as neo spiketrains. These are organized in the same way as the output of getContResps above and have the same input arguments.
  2. **getPulseResps:** Given a processed NIX File, the function retrieves the response traces to pulse train stimuli as neo analog signals organized in nested dictionaries. The keys at the first level are pulse duration and pulse interval values packed into tuples. The keys at the second level are trial numbers (starting with 0) and the keys at the third level belong to the set (“BeforeStimulus” , “DuringStimulus”, “AfterStimulus”) (see getContResps above for definitions).

Note that this function has input arguments for filtering the set of responses retrieved. The argument “pulsePars” can be used to filter responses based on pulse parameters (formated as a tuple: (<Pulse Duration>, <Pulse Interval>)) and “types” can used to specify the time intervals among “BeforeStimulus” , “DuringStimulus”, “AfterStimulus” that are fetched. Not specifying any arguments will fetch responses for all time intervals and pulse parameters.

- 1. **getPulseSpikes:** Given a processed NIX file, this function retrieves the spike times detected during responses to pulse stimuli as neo spiketrains. They are organized in the same way as the output of getPulseResps above and have the same input arguments.

# Usage

1. Here are the steps for using the data processing pipeline.

## Step 1: Installation

The first step towards using the data processing pipeline is installing the python package “GJEphys” and creating a python environment to run the code. The package code is hosted on Github (<https://github.com/wachtlerlab/GJEphys>). The procedure to complete the installations is as follows:

1. clone the package repository to a local machine as follows:

*git clone* [*https://github.com/wachtlerlab/GJEphys.git*](https://github.com/wachtlerlab/GJEphys.git)

1. Create a new Python 2.7 environment using conda (https://www.anaconda.com/what-is-anaconda/), pipenv (https://docs.pipenv.org/) or any other environment managers for python. NOTE: GJEphys only runs on python v2.7.
2. Activate the newly created environment
3. Install the python package cloned above

*pip install <path to the cloned directory “GJEphys”>*

## Step 2: Data preparation

The next step is arranging the input SMR and excel files on the file system as expected by the package GJEphys. The paths where input files are expected package are stored in variables of the file “GJEphys/GJEphys/folderDefs.py”. Look at this file for descriptions of the variables. The folders to which “NIXPath” and “spike2Path” point need to be create if they do no exist. The excel file containing all the metadata (“neuron_database.xlsx”) needs to be copied to the path to which the variable “excel” points. The name of the excel sheet containing the metadata of all experiments needs to be assigned to the variable “excelSheet”. All the experimentally generated SMR files which conform to the specifications in Fig.1 need to be copied into the folder to which the variable “spike2Path” points.

## Step 3: Importing

The next step is importing the data from smr files to NIX files using the script “GJEphys/scripts/usedInPubs/rawDataImporter.py”. The usage notes for this script is contained at the top of the file.

## Step 4: Processing

The next step is processing the imported NIX files to identify, tag and classify stimulus epochs as well as detect spikes. This is done using the script “GJEphys/scripts/usedInPubs/rawDataProcessor.py”. The usage notes for this script is contained at the top of the file

## Step 5: Fetching

The next step is fetching the data required for analysis using the methods of the class “RawDataAnalyzer” in “GJEphys/GJEphys/rawDataAnalyze.py”. The usage notes of the class and its methods are contained in the file.

# Use Cases

1. Following are some cases for the data processing pipeline.

## Continuous response stats

This use case was implemented in the script “GJEphys/scripts/useful/contStimStats.py”. The script reads the metadata excel file and generates a table summarizing the number of trials and number of experiments for every category of neuron and for every frequency of stimulus applied. An example of the output generated by this script is available on owncloud at https://filebox.g-node.org/owncloud/index.php/apps/files/ajax/download.php?dir=%2FGinJang_data%2FGinJang_Analysis%2FEphysAnalysis%2FAjay&files=contStimStats.pdf.

## Continuous response images

This use case was implemented in the script “GJEphys/scripts/useful/makeContStimImages.py”. This scripts uses a dictionary with neuron categories as keys and lists of experiment IDs as values. This dictionary is specified in the initial portion of the script. The script generates images depicting individual trials of continuous stimulus application and organizes them in folders. It addition, it generates overview images for every stimulus frequency used in an experiment. An example output of this script is available on owncloud: <https://filebox.g-node.org/owncloud/index.php/apps/files/?dir=%2FGinJang_data%2FGinJang_Analysis%2FEphysAnalysis%2FAjay%2FContStimsCatalogue>.

## Pulse response stats

This use case was implemented in the script “GJEphys/scripts/useful/pulseStimStats.py”. The script reads the metadata excel file and generates a table summarizing the number of trials and number of experiments for every category of neuron and for combination of pulse parameters used. An example of the output generated by this script is available on owncloud at https://filebox.g-node.org/owncloud/index.php/apps/files/ajax/download.php?dir=%2FGinJang_data%2FGinJang_Analysis%2FEphysAnalysis%2FAjay&files=pulseStimStats.pdf.

## Pulse response images

This use case was implemented in the script “GJEphys/scripts/useful/makePulseStimImages.py”. This scripts uses a dictionary with neuron categories as keys and lists of experiment IDs as values. This dictionary is specified in the initial portion of the script. The script generates images depicting individual trials of pulse-train stimulus application and organizes them in folders. It addition, it generates overview images for every combination of pulse parameters used in an experiment. An example output of this script is available on owncloud: https://filebox.g-node.org/owncloud/index.php/apps/files?dir=/GinJang_data/GinJang_Analysis/EphysAnalysis/Ajay/PulseStimsCatalogue

## Comparing Firing Rates and spike times

This use case was implemented in the script “/GJEphys/scripts/usedInPubs/compareFRSpikeTimes/compareFRSpikeTimes.py”. This was implemented primarily to analyze the data of DL-Int-1 neurons and for comparing features between newly emerged adult and forager DL-Int-1. The file contains several functions, some of which can be used for:

1. extracting firing rate and spike timing data of a specified set of experiments from corresponding processed NIX Files
2. Generating a plot comparing firing rates between neurons of different labor states
3. Generating a plot comparing spike timings between neurons of different labor states

## Comparing Total Number of spikes vs frequency

TODO

## PSTH of pulse train stimulus trials

This use case was implemented in the script “GJEphys/scripts/useful/pulseStimPSTH.py” and figures generated from this script are available on owncloud in the file: <https://filebox.g-node.org/owncloud/index.php/apps/files/ajax/download.php?dir=%2FGinJang_data%2FGinJang_Analysis%2FEphysAnalysis%2FAjay&files=SpikeLatency_trainOfPulses.pdf>

This script generates raster and PSTH plots of pulse train stimuli by identifying and time-aligning responses for those intervals for which sinusoidal vibration was applied during pulse stimuli.

## PSTH of continuous stimulus trials

This use case was implemented in the file “GJEphys/scripts/usedInPubs/contStimPSTH.py”. This file contains functions for

1. saving spike times of a specified set of experiments
2. estimate smoothed firing rate profiles from the saved spikes after time-aligning them based on stimulus onset.
