# Supplementary material for: Adaptations during Maturation in an Identified Honeybee Interneuron Responsive to Waggle Dance Vibration Signals
Source: eNeuro. 2019 Sep 5;6(5):ENEURO.0454-18.2019. doi: 10.1523/ENEURO.0454-18.2019 (PMC6731536; doi:10.1523/ENEURO.0454-18.2019)
Supplement: Table 1-1 — Summary statistics of 19 scalar morphometric measures applied to the whole arborization subregion of DL-Int-1 morphologies. The triplets in columns two and three represent minimum, median, and maximum values. Column four contains p values calculated using Mann–Whitney U test for differences between newly emerged adults and foragers. Measures with p values <5% are highlighted in red. Download Table 1-1, DOC file. [file sup_enu-eN-NWR-0454-18-s06.doc]

| **Measure** | **Newly emerged** | **Forager** | **P-Value** |
| --- | --- | --- | --- |
| Width (along X) (μm) | 170, 253, 293 | 238, 268, 299 | 0.2403 |
| Depth (along Z) (μm) | 176, 241, 258 | 193, 235, 248 | 0.8182 |
| Height (along Y)(μm) | 244, 308, 341 | 287, 333, 340 | 0.3939 |
| Avg. diameter (μm) | 0.99, 1.28, 1.45 | 1.18, 1.27, 1.41 | 0.8182 |
| Total dendritic length (x104 μm) | 1.24, 1.89, 3.32 | 1.11, 2.09, 2.48 | 0.9372 |
| Total dendritic surface (x104 (μm)2) | 5.05, 6.47, 15.4 | 3.92, 7.77, 10.8 | 1 |
| Total dendritic volume (x104 (μm)3) | 1.74, 2.17, 6.61 | 1.22, 2.69, 4.25 | 0.9372 |
| Total number of bifurcations | 383, 563, 1360 | 287, 571, 835 | 0.5887 |
| Max. Euclidean distance from root (μm) | 225, 269, 359 | 235, 259, 309 | 0.6991 |
| Max. path length from root (μm) | 366, 600, 719 | 485, 518, 617 | 0.5887 |
| Max. centrifugal order | 22, 45.5, 64 | 25, 35, 45 | 0.3095 |
| Avg. Burke taper | -0.375, -0.284, -0.118 | -0.396, -0.278, -0.099 | 0.8182 |
| Avg. contraction | 0.842, 0.859, 0.876 | 0.846, 0.872, 0.885 | 0.3095 |
| Avg. bifurcation angle (local) (degrees) | 118, 122, 124 | 119, 123, 126 | 0.3939 |
| Avg. bifurcation angle (remote) (degrees) | 98.4, 100, 103 | 94.4, 99.9, 103 | 0.4848 |
| Avg. partition asymmetry | 0.597, 0.611, 0.653 | 0.561, 0.576, 0.636 | 0.04113 |
| Avg. parent daughter diameter ratio | 0.975, 0.985, 0.995 | 0.972, 0.983, 0.992 | 0.4848 |
| Avg. sibling diameter ratio | 1.11, 1.12, 1.13 | 1.1, 1.12, 1.13 | 0.8182 |
| Hausdorff fractal dimension | 1.38, 1.42, 1.6 | 1.26, 1.38, 1.45 | 0.2403 |
