# Supplementary material for: Adaptations during Maturation in an Identified Honeybee Interneuron Responsive to Waggle Dance Vibration Signals
Source: eNeuro. 2019 Sep 5;6(5):ENEURO.0454-18.2019. doi: 10.1523/ENEURO.0454-18.2019 (PMC6731536; doi:10.1523/ENEURO.0454-18.2019)
Supplement: Table 1-2 — Summary statistics of 19 scalar morphometric measures applied to the main branch subregion of DL-Int-1 morphologies. The triplets in columns two and three represent minimum, median, and maximum values. Column four contains p values calculated using Mann–Whitney U test for differences between newly emerged adults and foragers. Measures with p values <5% are highlighted in red. It was not possible to calculate statistics for some measures (marked N/A) as one or more morphologies of newly emerged adult or forager DL-Int-1 neurons had no bifurcations in the main branch. Download Table 1-2, DOC file. [file sup_enu-eN-NWR-0454-18-s07.doc]

| **Measure** | **Newly emerged** | **Forager** | **P-Value** |
| --- | --- | --- | --- |
| Width (along X) (μm) | 10.4, 22.1, 26.5 | 25.2, 34.1, 61.4 | 0.004329 |
| Depth (along Z) (μm) | 7.7, 17.2, 54.2 | 15.3, 21.9, 33.2 | 0.5887 |
| Height (along Y)(μm) | 21.1, 33.3, 88.2 | 26.7, 31, 47.8 | 0.9372 |
| Avg. diameter (μm) | 2.02, 2.63, 2.86 | 1.95, 3.11, 3.8 | 0.2403 |
| Total dendritic length (x104 μm) | 0.00472, 0.00627, 0.0134 | 0.00659, 0.0093, 0.0135 | 0.09307 |
| Total dendritic surface (x104 (μm)2) | 0.0347, 0.0543, 0.114 | 0.056, 0.0928, 0.118 | 0.09307 |
| Total dendritic volume (x104 (μm)3) | 0.0183, 0.0409, 0.0803 | 0.0301, 0.0829, 0.0967 | 0.04113 |
| Total number of bifurcations | 0, 0, 0 | 0, 0, 1 | N/A |
| Max. Euclidean distance from root (μm) | 23.2, 34.5, 92.9 | 28.7, 38, 53.4 | 0.8182 |
| Max. path length from root (μm) | 44.2, 55.3, 131 | 39.3, 68.1, 116 | 0.9372 |
| Max. centrifugal order | 0, 0, 0 | 0, 0, 1 | N/A |
| Avg. Burke taper | -0.0585, -0.0332, 0.0242 | -0.479, 0.00273, 0.0787 | 0.2403 |
| Avg. contraction | 0.492, 0.697, 0.754 | 0.538, 0.816, 0.926 | 0.1797 |
| Avg. bifurcation angle (local) (degrees) | N/A | N/A | N/A |
| Avg. bifurcation angle (remote) (degrees) | N/A | N/A | N/A |
| Avg. partition asymmetry | N/A | N/A | N/A |
| Avg. parent daughter diameter ratio | 0.992, 0.999, 1 | 0.977, 0.997, 1 | N/A |
| Avg. sibling diameter ratio | N/A | N/A | N/A |
| Hausdorff fractal dimension | 0.949, 0.979, 1.04 | 0.995, 1.01, 1.06 | N/A |
