# Supplementary material for: Adaptations during Maturation in an Identified Honeybee Interneuron Responsive to Waggle Dance Vibration Signals
Source: eNeuro. 2019 Sep 5;6(5):ENEURO.0454-18.2019. doi: 10.1523/ENEURO.0454-18.2019 (PMC6731536; doi:10.1523/ENEURO.0454-18.2019)
Supplement: Table 1-3 — Summary statistics of 19 scalar morphometric measures applied to the dorsal branch subregion of DL-Int-1 morphologies. The triplets in columns two and three represent minimum, median, and maximum values. Column four contains p values calculated using Mann–Whitney U test for differences between newly emerged adults and foragers. Measures with p values <5% are highlighted in red. Download Table 1-3, DOC file. [file sup_enu-eN-NWR-0454-18-s08.doc]

| **Measure** | **Newly emerged** | **Forager** | **P-Value** |
| --- | --- | --- | --- |
| Width (along X) (μm) | 165, 220, 251 | 210, 259, 299 | 0.09307 |
| Depth (along Z) (μm) | 149, 187, 255 | 190, 228, 248 | 0.3095 |
| Height (along Y)(μm) | 152, 236, 263 | 212, 268, 294 | 0.04113 |
| Avg. diameter (μm) | 0.957, 1.25, 1.38 | 1.16, 1.25, 1.33 | 1 |
| Total dendritic length (x104 μm) | 0.883, 1.32, 2.58 | 0.858, 1.75, 2.06 | 0.6991 |
| Total dendritic surface (x104 (μm)2) | 3.32, 4.54, 11 | 3, 6.37, 8.56 | 0.8182 |
| Total dendritic volume (x104 (μm)3) | 1.05, 1.45, 4.21 | 0.928, 2.15, 3.33 | 0.6991 |
| Total number of bifurcations | 283, 445, 1.03e+03 | 225, 507, 735 | 0.8182 |
| Max. Euclidean distance from root (μm) | 143, 206, 306 | 187, 206, 222 | 0.9372 |
| Max. path length from root (μm) | 356, 481, 642 | 354, 504, 596 | 0.8182 |
| Max. centrifugal order | 22, 45.5, 64 | 25, 34.5, 44 | 0.3095 |
| Avg. Burke taper | -0.465, -0.333, -0.123 | -0.672, -0.316, -0.087 | 1 |
| Avg. contraction | 0.848, 0.857, 0.875 | 0.845, 0.871, 0.884 | 0.3939 |
| Avg. bifurcation angle (local) (degrees) | 118, 121, 123 | 118, 123, 125 | 0.4848 |
| Avg. bifurcation angle (remote) (degrees) | 99.3, 102, 103 | 95.6, 101, 104 | 0.5887 |
| Avg. partition asymmetry | 0.598, 0.618, 0.637 | 0.54, 0.582, 0.64 | 0.06494 |
| Avg. parent daughter diameter ratio | 0.975, 0.988, 0.993 | 0.972, 0.983, 0.993 | 0.6991 |
| Avg. sibling diameter ratio | 1.11, 1.12, 1.13 | 1.1, 1.12, 1.14 | 0.8182 |
| Hausdorff fractal dimension | 1.23, 1.38, 1.47 | 1.25, 1.33, 1.42 | 0.5887 |
