# Supplementary material for: Adaptations during Maturation in an Identified Honeybee Interneuron Responsive to Waggle Dance Vibration Signals
Source: eNeuro. 2019 Sep 5;6(5):ENEURO.0454-18.2019. doi: 10.1523/ENEURO.0454-18.2019 (PMC6731536; doi:10.1523/ENEURO.0454-18.2019)
Supplement: Table 1-4 — Summary statistics of 19 scalar morphometric measures applied to the ventral branch subregion of DL-Int-1 morphologies. The triplets in columns two and three represent minimum, median, and maximum values. Column four contains p values calculated using Mann–Whitney U test for differences between newly emerged adults and foragers. Measures with p values <5% are highlighted in red. Download Table 1-4, DOC file. [file sup_enu-eN-NWR-0454-18-s09.doc]

| **Measure** | **Newly emerged** | **Forager** | **P-Value** |
| --- | --- | --- | --- |
| Width (along X) (μm) | 139, 246, 293 | 206, 241, 269 | 0.9372 |
| Depth (along Z) (μm) | 101, 193, 258 | 149, 185, 220 | 0.9372 |
| Height (along Y)(μm) | 212, 252, 301 | 199, 273, 303 | 0.6991 |
| Avg. diameter (μm) | 1.16, 1.35, 1.75 | 1.18, 1.31, 1.64 | 0.9372 |
| Total dendritic length (x104 μm) | 0.2, 0.607, 1.38 | 0.243, 0.381, 0.505 | 0.2403 |
| Total dendritic surface (x104 (μm)2) | 0.714, 2.48, 6.3 | 0.87, 1.53, 2.55 | 0.3095 |
| Total dendritic volume (x104 (μm)3) | 0.232, 0.843, 2.48 | 0.263, 0.537, 1.11 | 0.3939 |
| Total number of bifurcations | 34, 156, 486 | 59, 73.5, 125 | 0.1275 |
| Max. Euclidean distance from root (μm) | 203, 237, 266 | 191, 228, 272 | 0.8182 |
| Max. path length from root (μm) | 294, 477, 598 | 299, 353, 396 | 0.3095 |
| Max. centrifugal order | 13, 30, 43 | 12, 16, 20 | 0.04383 |
| Avg. Burke taper | -0.182, -0.0654, -0.0259 | -0.14, -0.0648, -0.0313 | 0.9372 |
| Avg. contraction | 0.809, 0.868, 0.878 | 0.848, 0.872, 0.896 | 0.5887 |
| Avg. bifurcation angle (local) (degrees) | 117, 124, 127 | 119, 126, 131 | 0.5887 |
| Avg. bifurcation angle (remote) (degrees) | 81, 97.1, 102 | 86.3, 91, 97.3 | 0.3939 |
| Avg. partition asymmetry | 0.572, 0.599, 0.703 | 0.48, 0.589, 0.618 | 0.5887 |
| Avg. parent daughter diameter ratio | 0.962, 0.985, 1 | 0.97, 0.977, 0.992 | 0.4848 |
| Avg. sibling diameter ratio | 1.1, 1.13, 1.17 | 1.09, 1.12, 1.14 | 0.3095 |
| Hausdorff fractal dimension | 1.14, 1.24, 1.35 | 1.11, 1.14, 1.2 | 0.04113 |
